# Epstein-Barr virus in tumor-infiltrating B cells of myasthenia gravis thymoma: an innocent bystander or an autoimmunity mediator?

**DOI:** 10.18632/oncotarget.20731

**Published:** 2017-09-08

**Authors:** Paola Cavalcante, Stefania Marcuzzo, Sara Franzi, Barbara Galbardi, Lorenzo Maggi, Teresio Motta, Raffaella Ghislandi, Antonella Buzzi, Luisella Spinelli, Lorenzo Novellino, Fulvio Baggi, Carlo Antozzi, Fabio Conforti, Tommaso Martino De Pas, Massimo Barberis, Pia Bernasconi, Renato Mantegazza

**Affiliations:** ^1^ Neurology IV – Neuroimmunology and Neuromuscular Diseases Unit, Fondazione Istituto Neurologico “Carlo Besta”, 20133 Milan, Italy; ^2^ Department of Pathological Anatomy, ASST - Bergamo Est Ospedale Bolognini Seriate, 24068 Seriate Bergamo, Italy; ^3^ Department of General Surgery, ASST - Bergamo Est Ospedale Bolognini Seriate, 24068 Seriate Bergamo, Italy; ^4^ Unit of Sarcomas and Thymomas, European Institute of Oncology, 20136 Milan, Italy; ^5^ Histopathology and Molecular Diagnostics Unit, European Institute of Oncology, 20136 Milan, Italy

**Keywords:** autoimmunity, myasthenia gravis, Epstein-Barr virus, thymoma, toll-like receptors

## Abstract

The thymus plays a key role in myasthenia gravis (MG), a B cell-mediated autoimmune disorder affecting neuromuscular junction. Most MG patients have thymic abnormalities, including hyperplasia and thymoma, a neoplasm of thymic epithelial cells. Epstein-Barr virus (EBV) is associated with autoimmune diseases and tumors. Recently, we showed EBV persistence and reactivation in hyperplastic MG thymuses, suggesting that EBV might contribute to intra-thymic B cell dysregulation in MG patients. Here, we investigated EBV involvement in thymoma-associated MG, by searching for EBV markers in MG (n=26) and non-MG (n=14) thymomas. EBV DNA and EBV-encoded small nuclear RNA (EBER) 1 transcript were detected in 14/26 (53.8%) and 22/26 (84.6%) MG thymomas, and only in 3 of 14 (21.4%) non-MG thymomas. Latent EBNA2 and late gp350/220 lytic transcripts were undetectable in all, but one, thymomas, and early lytic BZLF1 transcript was absent in all samples, suggesting that early infection events and EBV reactivation were very rare in thymomas. EBER1 and 2-positive cells were detected in MG, but not in non-MG, thymomas, as well as cells expressing EBV latency proteins (EBNA1, LMP1, LMP2A), that were mainly of B cell phenotype, indicating EBV association with MG rather than with thymoma. Toll-like receptor (TLR) 3 transcriptional levels were higher in MG than non-MG thymomas and positively correlated with EBER1 levels, suggesting a role for EBERs in TLR3 activation. Our findings show that EBV is commonly present in thymoma-infiltrating B cells of myasthenic patients, indicating a contribution of EBV to B cell-mediated autoreactivity in MG associated with thymic tumor.

## INTRODUCTION

Myasthenia gravis (MG) is a prototypical antibody-mediated autoimmune disease affecting the neuromuscular junction, mainly caused by anti-acetylcholine receptor (AChR) autoantibodies, ultimately leading to skeletal muscle weakness and fatigability [1]. Pathological abnormalities of the thymus characterize most AChR-MG patients, and thymectomy may be associated with increased frequency of remission, thus suggesting thymus involvement in the onset and perpetuation of autoimmunity to the AChR [2–5]. MG-associated thymic abnormalities include hyperplasia, the most common alteration in early-onset (<50 years of age) MG patients, and thymoma, occurring most frequently in late-onset (>50 years) MG patients [2, 3, 6]. Thymomas are slow-growing, locally invasive neoplasms of thymic epithelial cells (TECs), associated with a variety of autoimmune diseases [7–9]; MG is present in approximately 30-45% of thymoma patients [9]. The World Health Organization (WHO) classification recognizes type A, AB, B1, B2 and B3 thymomas depending on lymphocyte content and epithelial cell features [10–12]. Type B2 thymoma is the histological diagnosis most frequently associated with MG, followed by types AB and B1 [6, 13, 14]. Apart from very rare exceptions, MG patients with thymoma have AChR antibodies, usually showing generalized muscle involvement with more severe disease, and are less responsive to treatment than those without thymoma, with lower rates of complete stable remission [6, 15, 16]. Antibodies reacting with muscle antigens including actin, myosin, titin and ryanodine receptor are also present in MG thymoma patients, but their role in the disease pathogenesis is unclear [1, 17]. ‘Dangerous’ autoantigen presentation in the context of autoimmune regulator (AIRE) deficiency, abnormal T cell selection, and failure in regulatory T cell (Treg) generation are postulated intra-tumorous mechanisms, not mutually exclusive, driving the autoimmune response in thymoma-associated MG [3, 16]. However, triggering factors leading to immune dysregulation and chronic autoimmunity in MG patients with thymoma are not known yet. Recently, an antiviral gene signature was identified in MG thymomas, characterized by increased expression of type I interferons (IFNs) and Toll-like receptor (TLR) 3, a TLR known to recognize viral double strand (ds) RNA molecules associated with replication of many viruses, thus suggesting that MG might develop after a pathogen infection in thymoma patients [18].

Epstein-Barr virus (EBV) is one of the most common viruses in humans, infecting >90% of the world population and establishing persistent latent infection in the host [19]. Most people become infected with EBV during childhood or adolescence and achieve adaptive immunity against the virus. Although EBV is harmless, in some cases its transforming capacity might promote malignant transformation or favor autoimmunity; indeed, EBV has been associated with several autoimmune diseases and cancers [20–22]. The life cycle of EBV includes a primary infection of naïve B cells followed by chronic persistence (or latency) of EBV in memory B cells. Markers of EBV replication and latency can be detected by sensitive molecular and immunohistochemistry techniques [23]. Recently, we provided evidence of EBV persistence and reactivation in B cells and plasma cells of hyperplastic and involuted thymuses from MG patients, but not in normal control thymuses, suggesting that the virus might be involved in the intra-thymic MG autoimmune process, likely through the activation and immortalization of autoreactive B cells and induction of pathogenic TLR7 and 9 signaling [24–26]. Whether EBV is also involved in MG associated with thymoma is not known. Previous studies designed to investigate the potential association between EBV and thymoma produced contrasting results [18, 27–31]. To better address this issue, we investigated the presence of EBV nucleic acids and proteins in MG and non-MG thymoma specimens by combining molecular, *in situ* hybridization and immunohistochemistry techniques.

## RESULTS

### High detection frequency of EBV DNA and EBER1 transcript in MG thymomas

The study included 26 MG thymoma and 14 non-MG thymoma patients (Table 1) and 6 non-pathological control thymuses obtained from cardiopathic patients during heart surgery.

**Table 1:** Summary of the main clinical characteristics of thymoma patients included in the study

Patients	Sex	Age at MG onset	Age at thymectomy	Autoantibodyspecificity	Pre-thymectomy therapy
MG (+) T1	M	>50	55	NA	NA
MG (+) T2	M	>50	53	A+^a^	Antiac+Cortic+Aza
MG (+) T3	M	<50	50	A+^a^	Antiac+Cortic
MG (+) T4	M	>50	51	NA	NA
MG (+) T5	F	<50	47	A+ R+^b^	Antiac+Cortic+Aza
MG (+) T6	F	<50	42	A+ T+ R+	Antiac+Aza
MG (+) T7	F	<50	43	A+ T+ R+	Antiac+Cortic+Aza
MG (+) T8	M	>50	53	A+^a^	Antiac
MG (+) T9	M	>50	63	A+^a^	Antiac
MG (+) T10	F	>50	75	A+^a^	Aza
MG (+) T11	F	<50	50	A+ R+^b^	Antiac+Cortic
MG (+) T12	F	<50	45	A+^a^	Antiac+Cortic+Aza
MG (+) T13	M	<50	23	A+^a^	Antiac+Cortic
MG (+) T14	M	>50	63	A+ T+ R+	Antiac+Cortic
MG (+) T15	F	>50	51	A+ R+^b^	Antiac
MG (+) T16	M	<50	26	A+ R+^b^	Antiac+Cortic
MG (+) T17	F	<50	35	A+ T+ R+	Antiac+Cortic+Aza
MG (+) T18	F	>50	67	A+^a^	Antiac+Cortic
MG (+) T19	F	>50	56	A+ T+ R-	Antiac+Cortic+Aza
MG (+) T20	M	>50	54	A+^a^	Antiac
MG (+) T21	F	>50	63	A+^a^	Antiac+Cortic+Aza
MG (+) T22	F	<50	33	A+^a^	Antiac+Cortic+Aza
MG (+) T23	F	<50	43	A+ R+^b^	Antiac
MG (+) T24	F	NA	53	A+ T+ R+G+^c^	Antiac+Cortic+Aza
MG (+) T25	F	>50	60	A+^a^	Antiac
MG (+) T26	F	<50	35	A+ T+ R+	Antiac+Cortic
MG (-) T1	F	-	76	-	-
MG (-) T2	F	-	40	-	-
MG (-) T3	M	-	42	-	-
MG (-) T4	F	-	61	-	-
MG (-) T5	F	-	20	-	-
MG (-) T6	F	-	51	-	-
MG (-) T7	M	-	78	-	-
MG (-) T8	M	-	69	-	-
MG (-) T9	M	-	39	-	-
MG (-) T10	F	-	44	-	-
MG (-) T11	M	-	65	-	-
MG (-) T12	M	-	47	-	-
MG (-) T13	F	-	72	-	-
MG (-) T14	M	-	44	-	-

MG (+) T1-26: thymomas from MG patients; MG (-) T1-14: thymomas from non-MG patients; A+: positive to anti-AChR antibodies; T+: positive to anti-titin antibodies; R+: positive to anti-ryanodine receptor antibodies; Antiac: anti-cholinesterase treatment; Cortic: corticosteroids; Aza: azathioprine; NA: not available.

^a^Data on anti-titin and anti-ryanodine receptor antibodies are not available.

^b^Anti-titin antibodies were negative.

^c^MG (+) 24 thymoma was from a patient positive for anti-glutamic acid decarboxylase antibodies (G), along with anti-AChR, anti-titin and anti-ryanodine receptor antibodies.

**Figure 1:**
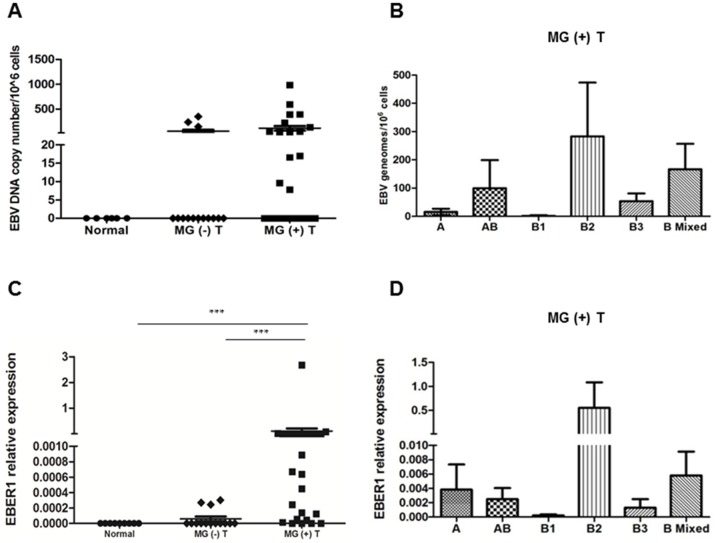
High detection frequency of EBV genome and non-coding EBER1 transcript in MG thymomas **(A)** Results of quantitative real-time PCR analysis to assess EBV DNA load in normal thymuses (n=6), MG (-) (n=14) and MG (+) (n=26) thymomas (T). In the graph, viral load data are expressed as copy number of EBV genomes per 1 million cells. Each point corresponds to a sample and the horizontal line indicates the mean value of EBV DNA load with SEM for each sample group. **(B)** EBV DNA load values in MG (+) thymomas grouped according to the WHO histological types. **(C)** Results of real-time PCR analysis to assess EBER1 expression in normal thymuses (n=6), MG (-) (n=14) and MG (+) (n=26) thymomas (T). In the graph, EBER1 levels are expressed as relative values (2^-ΔCt^ × 100) normalized to the housekeeping gene 18S. Each point corresponds to a sample and the horizontal line indicates the mean EBER1 expression level with SEM for each sample group. P values were assessed by Kruskal-Wallis test with Bonferroni post-hoc test, ^***^p <0.001. **(D)** EBER1 relative expression values in MG (+) thymomas grouped according to the WHO histological types.

To determine the EBV DNA load in MG and non-MG thymomas, we used a quantitative real-time PCR method (Supplementary Figure 1) based on the simultaneous amplification of the EBV DNA polymerase (Pol) and the human beta 2 microglobulin (β2m) genes [32]. EBV DNA was detected in 3/14 (21.4%) non-MG thymomas and 14/26 (53.8%) MG thymomas. Viral load, expressed as number of EBV DNA copies per 10^6^ cells, ranged from 7.8 to 985.5 copies in the MG thymoma group, and from 145.4 to 352.0 in non-MG thymomas (Table 2, Figure 1A). In both the MG thymomas and non-MG thymomas, the highest EBV DNA load mean values were observed in the WHO types B2 and mixed B2/B3 (Table 2, Figure 1B). As expected, mediastinal B cell lymphoma and Hodgkin's lymphoma samples, analyzed as positive controls, were highly positive for EBV DNA, with a viral load of 4,500 and 1,500 EBV DNA copies per 10^6^ cells. EBV DNA was undetected in non-pathological control thymuses (Figure 1A), and in pleural fibrous tumor and Jurkat T cells, analyzed as negative controls (not shown).

**Table 2:** EBV DNA load and EBER1 detection in MG and non-MG thymomas

PATIENTS	EBV DNA load(EBV genomes /10^6 cells)^a^	EBER1 transcript^b^	WHO histological type
MG (+) T1	9.6	+	A
MG (+) T2	37.4	+	A
MG (+) T3	Und	Und	A
MG (+) T4	597.2	+	AB
MG (+) T5	Und	+	AB
MG (+) T6	Und	+	AB
MG (+) T7	Und	Und	AB
MG (+) T8	Und	Und	AB
MG (+) T9	Und	+	AB
MG (+) T10	Und	+	B1
MG (+) T11	7.8	+	B1
MG (+) T12	Und	Und	B1
MG (+) T13	Und	+	B1
MG (+) T14	391.0	+	B2
MG (+) T15	Und	+	B2
MG (+) T16	Und	+	B2
MG (+) T17	38.2	+	B2
MG (+) T18	985.5	+	B2
MG (+) T19	16.6	+	B3
MG (+) T20	47.3	+	B3
MG (+) T21	17.0	+	B3
MG (+) T22	133.6	+	B3
MG (+) T23	47.0	+	B1/B2
MG (+) T24	394.5	+	B1/B2
MG (+) T25	Und	+	B2/B3
MG (+) T26	226.0	+	B2/B3
MG (-) T1	Und	Und	A
MG (-) T2	Und	Und	AB
MG (-) T3	Und	Und	AB
MG (-) T4	Und	Und	AB
MG (-) T5	Und	Und	B1
MG (-) T6	145.2	+	B2
MG (-) T7	Und	Und	B2
MG (-) T8	Und	Und	B3
MG (-) T9	Und	Und	B3
MG (-) T10	238.6	+	B3
MG (-) T11	Und	Und	B1/B2
MG (-) T12	Und	Und	B2/B3
MG (-) T13	Und	Und	B2/B3
MG (-) T14	352.0	+	B2/B3

MG (+) T1-26: thymomas from MG patients; MG (-) T1-14: thymomas from non-MG patients.

^a^EBV DNA load, expressed as number of EBV genome copies per 1 million cells of thymoma tissue, was determined by real-time PCR for the EBV polymerase gene, via comparison with a standard curve obtained by serial dilution of DNA from EBV-positive Namalwa cells (diploid line containing two integrated EBV genomes).

^b^Detection of EBV-encoded small RNA 1 (EBER1) transcript by real-time PCR.

+: Detected; Und: undetected.

EBV-encoded small RNA (EBER) 1, the most abundant viral transcript expressed during EBV latency along with EBER2 [33], was also investigated in the same samples. Most MG thymomas (22/26, 84.6%) were positive for EBER1, whereas among non-MG thymomas it was detected only in the three samples out of 14 (21.4%) positive for EBV DNA (Table 2, Figure 1C). As expected, EBER1 was detected in the mediastinal B cell lymphoma and Hodgkin's lymphoma samples, whereas it was undetectable in normal control thymuses, pleural tumor and Jurkat T cells. A representative EBER1 amplification curve in positive and negative samples is provided in Supplementary Figure 2. Transcriptional levels of EBER1 were significantly higher in MG (0.108 ± 0.103) compared to non-MG thymomas (0.000058 ± 0.00011, p<0.001); the highest relative expression values were observed in WHO type B2 and mixed B2/B3 MG thymomas (Figures 1C, 1D). In the MG thymoma group, EBER1-positive samples were more numerous than samples positive for EBV DNA (22 versus 14); moreover, all MG and non-MG samples positive for EBV DNA were also positive for EBER1, but we did not observe samples negative for EBER1 that were positive for EBV DNA (Table 2). This might be due to the sensitivity of the real-time PCR methods, that could be higher for the amplification of EBER1 than for EBV genome, since EBER1 molecule is present in multiple copies in cells latently infected by EBV [33]. The frequency of EBER1 detection was significantly higher in the group of MG thymomas versus non-MG thymomas (odd ratio: 20.2; p< 0.0001), suggesting EBV association with MG rather than thymoma.

EBV DNA load values and EBER1 levels did not significantly differ between MG patients untreated and treated with corticosteroids before thymectomy, alone or in combination with azathioprine (data not shown), suggesting that EBV presence in MG thymomas is not influenced by immunosuppressive therapy.

### Absence of latent EBNA2, early BZLF1 and late gp350/220 lytic EBV transcripts in MG and non-MG thymomas

EBV nuclear antigen (EBNA) 2 is the first latency protein synthesized after EBV infection of naïve B cells; its expression reflects the initial stage of EBV infection [34]. We analyzed the expression of EBNA2 transcript in MG and non-MG thymomas by real-time PCR. All the examined MG and non-MG samples, except for one MG thymoma [MG (+) T24], were negative for EBNA2 transcript, which was also undetected in the pleural fibrous tumor and normal thymuses. As expected, EBNA2 was detected in EBV-positive mediastinal B cell lymphoma and JY cells.

To assess EBV reactivation, we analyzed BZLF1 (early lytic) and gp350/220 (late lytic) gene expression by nested RT-PCR and real-time PCR. BZLF1 encodes a transactivator protein regulating expression of early lytic genes, whereas gp350/220, also known as BLLF1, encodes a glycoprotein of the EBV capsid [35]. BZLF1 mRNA was detected in Namalwa and JY lymphoid cell lines and Hodgkin's lymphoma, but in none of the examined MG and non-MG thymomas and normal thymuses (Supplementary Figure 3). The late gp350/220 lytic transcript was also detected in positive control cells and Hodgkin's lymphoma, but it was absent in all the examined MG, non-MG samples and normal thymuses, except in the MG thymoma [MG (+) T24], that was positive also for EBNA2 mRNA. These data suggest that EBV reactivation and early infection events are absent or very rare in MG and non-MG thymomas.

### Detection of EBERs in MG thymomas by *in situ* hybridization

**Figure 2:**
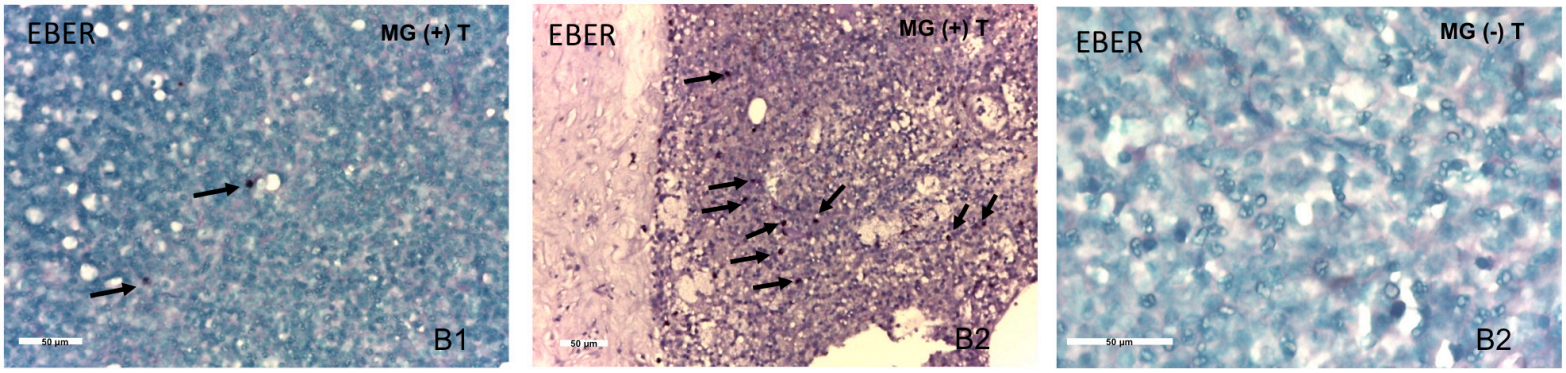
Detection of EBV-encoded small RNAs (EBERs) in MG thymomas, but not in non-MG thymomas, by *in situ* hybridization Left and middle panels show low and high density of EBER-positive cells (arrows, blue-black nuclei) in the thymomatous tissue of MG patients [MG (+) T11 (type B1) and T14 (type B2), respectively]. The right panel shows absence of EBER-positive cells in the thymomatous tissue of a non-MG thymoma [MG (-) T7 (type B2)].

*In situ* hybridization to detect EBERs (EBER1 and 2) was performed in 9 MG and 5 non-MG thymoma samples, for which formalin-fixed paraffin-embedded thymoma and non-tumoral adjacent thymic tissue blocks were available. No EBER signal was found in the tumoral tissue or in the non-tumoral adjacent thymic tissues from non-MG thymomas (Figure 2). The synchronous pleural metastases analyzed in 2 of the 5 non-MG thymomas were also negative for EBERs (not shown). By contrast, variable number of cells positive for EBERs was detected in 5 of the 9 (55.6%) tumoral tissues of MG thymomas (Figure 2), in which non-tumoral adjacent tissue was negative (not shown). All MG thymomas positive for cells expressing EBERs were also positive for EBER1 transcript (Supplementary Table 1); moreover, among the examined MG thymomas, the highest density of EBER-positive cells was found in the type B2 MG sample showing the highest EBV DNA load value and also the highest EBER1 expression levels [MG (+) T14] (Supplementary Table 1, Figure 2). The 6 non-pathological control thymuses, the same analyzed here for EBV DNA and EBER1 by real-time PCR, were previously tested by *in situ* hybridization for EBERs and resulted negative [24].

### Increased frequency of intra-tumoral B cells and EBNA1-positive cells in MG thymomas

**Figure 3:**
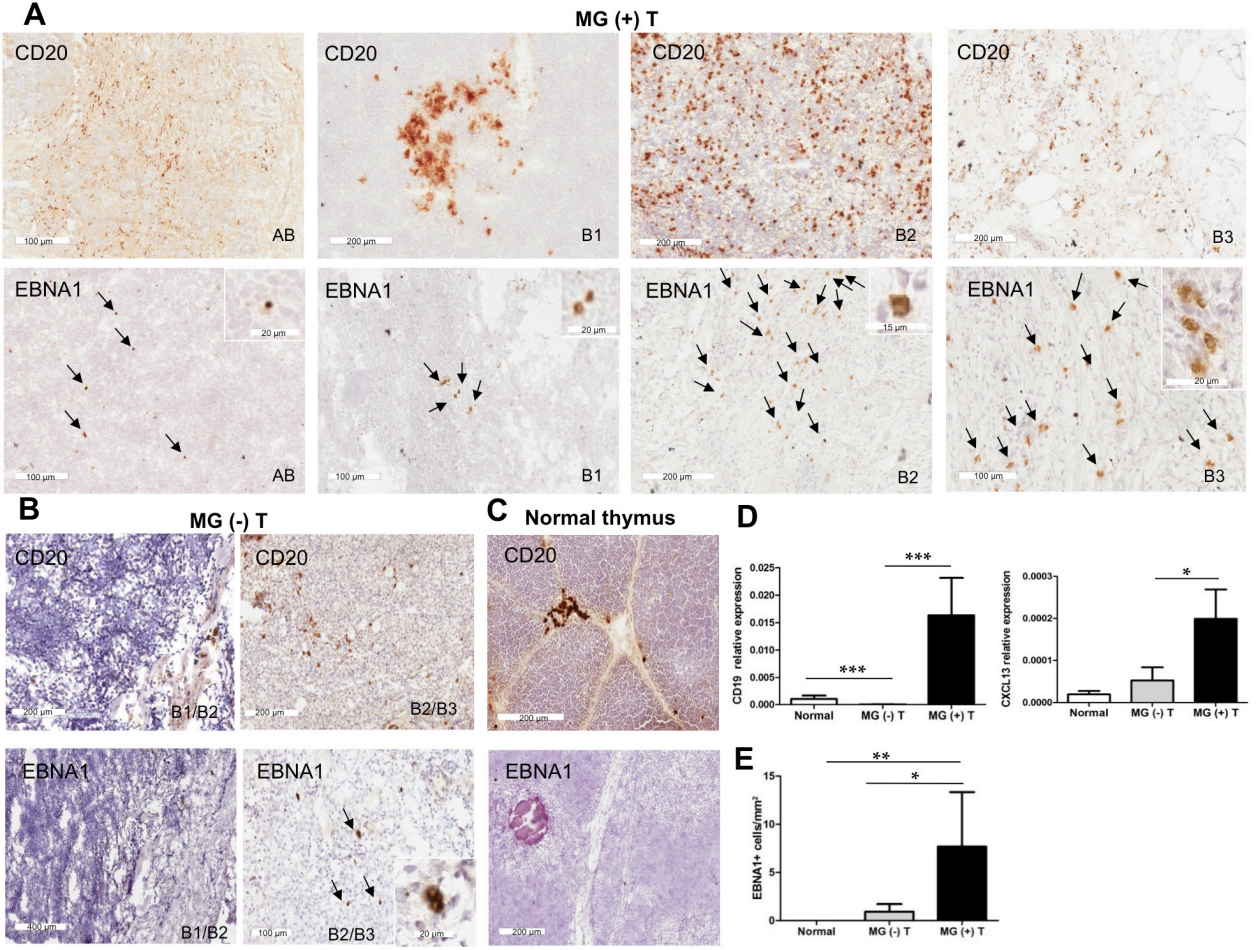
High degree of B cell infiltration and detection of EBNA1-positive cells in thymomas associated with MG **(A)** Immunohistochemistry analysis showing the presence of numerous CD20-positive B cells (upper panels) and EBNA1-positive cells (arrows, lower panels) in MG thymomas of different WHO types [from left to right MG (+) T7 (type AB), T11 (type B1), T17 (type B2) and T20 (type B3)]. The insets in the lower panels show enlargement of EBNA1-positive cells in the same thymoma tissues represented in the main panels, displaying the nuclear localization of EBNA1. **(B)** Immunohistochemistry analysis showing absent or few CD20-positive B cells (upper panels) and EBNA1-positive cells (arrows, lower panels) in non-MG thymomas [MG (-) T11 (type B1/B2) and MG (-) T14 (type B2/B3)]. The inset in the lower right panel shows enlargement of EBNA1-positive cells present in the same thymoma tissue represented in the main panel. **(C)** Immunolocalization of CD20-positive cells (upper panel) and absence of EBNA1-positive cells (lower panel) in normal thymus sections from a 22 year-old woman. **(D)** Results of real-time PCR analysis to assess mRNA levels of CD19, a B cell marker, and CXCL13, a B cell-attracting chemokine, in normal thymuses (n=6), MG (-) (n=14) and MG (+) (n=26) thymomas (T). The graphs show the mean (± SEM) of CD19 (left) and CXCL13 (right) mRNA levels, expressed as relative values (2^-ΔCt^ × 100) normalized towards the housekeeping gene 18S, in each sample group. **(E)** Mean number (± SEM) of EBNA1-positive cells per thymoma area (mm^2^) obtained in each sample group. P values were assessed by Kruskal-Wallis test with Bonferroni post-hoc test, ^***^p<0.001, ^**^ p<0.01, ^*^p<0.05.

Considering the EBV tropism for B cells [20], we analyzed their frequency in thymomas from MG and non-MG patients. By immunohistochemistry, we found an increased proportion of infiltrating CD20-positive B cells in MG thymomas compared to non-MG thymomas and normal thymuses (Figures 3A-3C). In all the MG thymomas analyzed, particularly in WHO B types, B cells were highly diffused throughout the neoplastic tissue, or present in the form of small or large aggregates in the connective tissue (Figure 3A, Supplementary Figure 4). By contrast, in non-MG thymomas, B cells were occasionally detected, mainly as isolated cells or small aggregates (Figure 3B, Supplementary Figure 4). These results were confirmed at the molecular level by the observation of higher transcriptional levels of the B cell marker CD19 in MG thymomas, compared with non-MG thymomas and normal thymuses (Figure 3D). The C-X-C motif chemokine ligand 13 (CXCL13), a B cell attracting chemokine known to mediate the homing and motility of B cells in lymphoid tissues [36], was also up-regulated in MG compared with non-MG thymomas, suggesting that the higher degree of B cell infiltration in thymomas from MG patients could be due to CXCL13 overexpression (Figure 3D).

Cells positive for the latent EBNA1 protein were commonly observed in MG tumor tissues (12/12) (Figure 3A; Supplementary Figure 4), whereas in non-MG thymomas they were only detected in the three cases previously positive for EBV DNA and EBER1 transcript [MG (-) T6, 10, and 14] (Figure 3B, Supplementary Figure 4). MG thymomas showed variable numbers of cells expressing EBNA1, with high density of positive cells in the neoplastic tissue of thymomas with high B cell content, particularly B2 and B3 thymomas (Figure 3A, Supplementary Figure 4). The number of EBNA1-positive cells normalized per thymoma area was significantly higher in MG than in non-MG thymomas (p<0.05, Figure 3E). EBNA1-positive cells were detected in Hodgkin's lymphoma, but not in pleural tumor tissue sections (Supplementary Figure 4) and normal thymuses (Figures 3C, 3E).

### Detection of B cells positive for the latency EBV proteins LMP1 and LMP2A in MG thymomas

**Figure 4:**
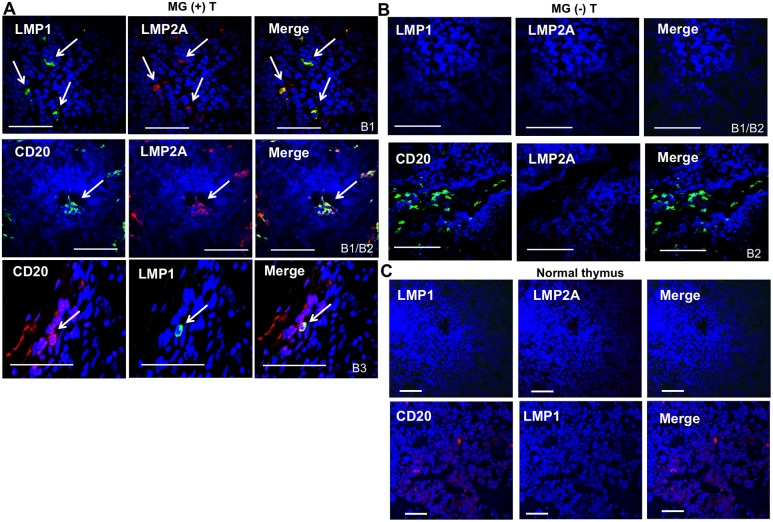
High detection frequency of latency phase EBV proteins, LMP1 and LMP2A, in CD20-positive B cells of thymomas associated with MG **(A)** Double immunofluorescence stainings of MG-associated thymoma sections for: LMP1 (green) and LMP2A (red), upper panels [MG (+) T11 (type B1)]; CD20 (green) and LMP2A (red), middle panels [MG (+) T24 (type B1/B2)]; CD20 (red) and LMP1 (green) lower panels [MG (+) T20 (type B3)]. Arrows in each panel show double positive cells present in the neoplastic MG tissue as isolated cells or small aggregates. **(B)** Double immunofluorescence staining of non-MG thymoma sections for: LMP1 (green) and LMP2A (red), upper panels [MG (-) T11 (type B1/B2)]; CD20 (green) and LMP2A (red), lower panels [MG (-) T6 (type B2)]. **(C)** Absence of positive signals for LMP1 and LMP2A in normal thymuses. Upper panels show double immunofluorescence stainings of normal thymic sections from a 17-year-old man for LMP1 (green) and LMP2A (red); lower panels show double immunofluorescence stainings of sections from the same thymus for CD20 (red) and LMP1 (green). Blue staining in A, B e C panels shows DAPI-positive nuclei. Magnification bars: 50 μm.

**Figure 5:**
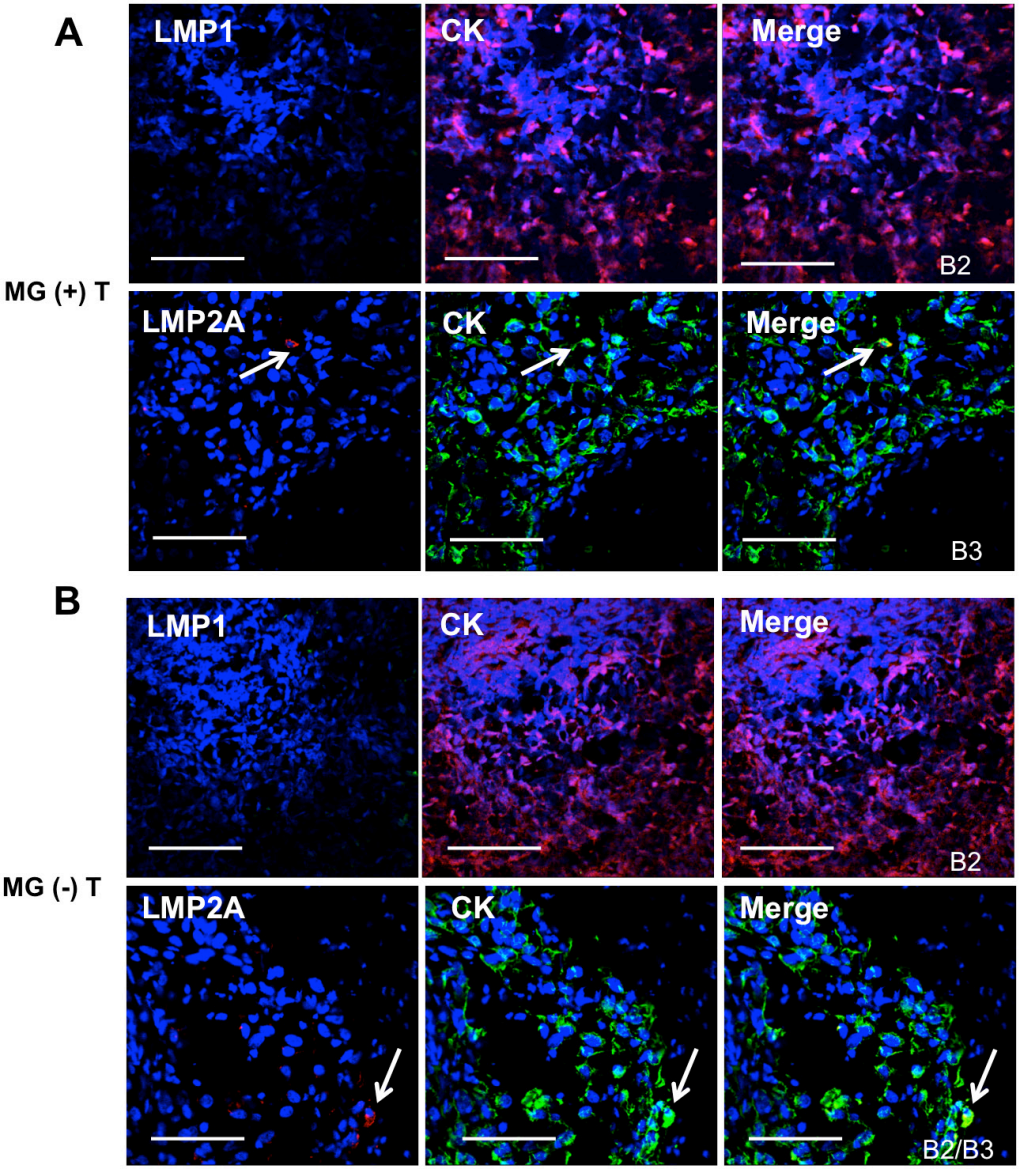
Absence or very rare detection of latency phase EBV proteins, LMP1 and LMP2A, in thymic epithelial cells of MG and non-MG thymomas **(A)** Double immunofluorescence stainings of MG-associated thymoma sections for: LMP1 (green) and cytokeratin (CK, red), marker of epithelial cells, upper panels [MG (+) T17 (type B2)]; LMP2A (red) and CK (green), lower panels [MG (+) T20 (type B3)]. The arrow in the lower panels indicates the presence of a single CK-positive cells expressing LMP2A. **(B)** Double immunofluorescence stainings of non-MG thymoma sections for: LMP1 (green) and CK (red), upper panels [MG (-) T6 (type B2)]; LMP2A (red) and CK (green), lower panels [MG (-) T14 (type B2/B3)]. The arrow in the lower panels indicates the presence of a single CK-positive cells expressing LMP2A. Blue staining in A and B panels shows DAPI-positive nuclei. Magnification bars: 50 μm.

To characterize the phenotype of EBV-infected cells, detected in MG thymomas by *in situ* hybridization for EBERs and EBNA1-specific immunostaining, we performed double immunofluorescence staining to localize the EBV latency membrane proteins LMP1 and LMP2A in B cells and TECs, the possible cell targets of EBV infection. Cells positive for both LMP1 and LMP2A were frequently found in the neoplastic tissue of MG thymomas, but not in non-MG thymomas and normal thymus (Figure 4). These cells showed mainly a B cell phenotype, as demonstrated by the observation that a proportion of infiltrating CD20-positive B cells, but only very rare cytokeratin (CK)-positive TECs, expressed the two latent EBV proteins in the MG thymomas examined (Figure 4A, Figure 5A). In MG thymomas, the mean percentages (± SD) of LMP2A/CD20 and LMP1/CD20 double positive cells, estimated on the total of CD20-positive cells per thymoma section, were 29.8 ± 14.1 and 16.8 ± 14.4, with the highest values being observed in B2 type thymomas. No signal for LMP1 and LMP2A was found in infiltrating B cells in non-MG thymomas (Figure 4B). CK/LMP1 double positive TECs were never detected in MG and non-MG thymomas (Figure 5), whereas CK/LMP2A double positive cells were only occasionally found in MG-associated thymomas (Figure 5A), whereas rare CK/LMP2A double positive TECs were found in one of the three non-MG thymomas, found positive for EBV DNA and EBER1 [MG (-) T14] (Table 2) (Figure 5B). Such evidence of absent or very rare EBV presence in TECs from MG thymomas was confirmed by real-time PCR analysis of EBER1 in cDNA samples of TEC lines isolated from three EBER1-positive MG thymomas [MG (+) T11, 14 and 16] (Table 2). Indeed, all the examined TEC lines resulted negative for EBER1 transcript (data not shown). LMP1/ and LMP2A/CD20 positive B cells were detected in high number in thymic sections of patients with mediastinal B cell lymphoma or Hodgkin's lymphoma, but not in pleural tumor tissue sections (Supplementary Figure 5).

Altogether, the present data show that infiltrating B cells positive for EBV, but not TECs, are commonly present in the neoplastic thymic tissue of thymoma patients with MG.

### Increased TLR3 transcript levels in MG thymomas: possible activation by EBERs?

**Figure 6:**
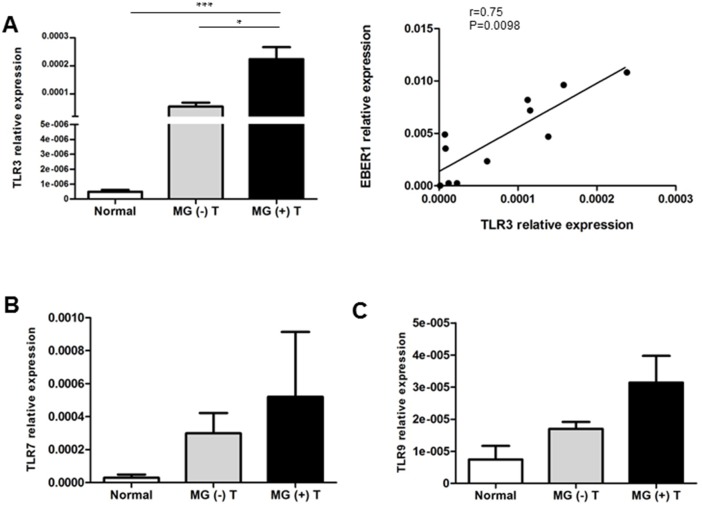
Expression of TLR3, 7 and 9 in MG and non-MG thymomas, and relationship between TLR3 mRNA increase and EBER1 expression in EBV-positive MG thymomas Results of real-time PCR analysis to assess TLR3 **(A)**, TLR7 **(B)** and TLR9 **(C)** in normal thymuses (n=6), MG (-) (n=14) and MG (+) (n=26) thymomas (T). In each graph, transcriptional levels of TLR3, 7 or 9 are expressed as mean relative values (2^-ΔCt^ × 100) ± SEM, normalized to the housekeeping gene 18S. P values were assessed by Kruskal-Wallis test with Bonferroni post-hoc test, ^***^p<0.001, ^*^p<0.05. The right graph in (A) shows positive correlation between TLR3 and EBER1 expression levels (Spearman test, r=0.75; p<0.01) in EBV-positive MG (+) thymomas.

Considerable evidence indicates that EBER release from EBV-infected cells may activate innate immune signaling by TLR3, which is known to recognize dsRNA [37, 38]. Recently, Cufi and collaborators demonstrated TLR3 overexpression in MG thymomas compared to normal thymuses, suggesting that TLR3-mediated pathways may be activated via pathogen-related dsRNAs in the neoplastic tissue of MG thymomas [18]. Here, we compared TLR3 expression between MG and non-MG thymomas, to verify whether TLR3 overexpression in MG thymomas was strictly linked to thymoma or instead was specific for MG. We confirmed increased transcriptional levels of TLR3 in MG thymomas compared with normal thymuses (Figure 5A) and, interestingly, we found that TLR3 was significantly overexpressed in MG-associated thymomas compared with thymomas from patients without MG (Figure 6A). We also observed that in EBV-positive MG thymomas TLR3 mRNA levels positively correlated with those of EBER1 (Spearman test, r=0.75; p<0.01, Figure 6A), suggesting a role for this viral small RNA molecule in stimulating TLR3 signaling and a contribution of TLR3 in MG associated with thymoma.

We also compared mRNA levels of TLR7 and TLR9, two endosomal-lysosomal receptors able to recognize bacterial or viral RNA and DNA [39] and found significantly increased in EBV-positive hyperplastic MG thymuses [26], among normal thymuses, MG and non-MG thymomas. We observed a slight, but not significant, increase of transcriptional levels of both receptors in MG thymomas compared with non-MG thymomas and normal thymuses (Figure 6B, 6C).

## DISCUSSION

Factors triggering B cell-mediated autoimmunity in MG are still not completely understood. MG complexity highlights the need to study distinct clinical subgroups to improve our knowledge of the causative agents and mechanisms leading to the disease [1]. A common feature of MG patients is the presence of pathologic alterations of the thymus, suggesting a pivotal role of this organ, which normally guarantees central self-tolerance, in development and maintenance of an autoantigen-specific antibody response [2, 3]. The presence of hyperplasia or thymoma in MG patients suggests different mechanisms of intra-thymic MG pathogenesis, although shared thymic dysregulation events in patients with different thymic alterations cannot be excluded. Chronic inflammation and innate immune activation, possibly linked to pathogen infections, are postulated mechanisms leading to anti-AChR autosensitization in non-thymomatous MG patients with thymic hyperplasia [40]. In thymoma-associated MG, the postulated intra-thymic alterations driving autoreactivity include an altered autoantigen presentation and a defective Treg compartment, which favor autoreactive T cell activation and failure of central tolerance [16]. These features are likely to explain the higher frequency of autoimmune phenomena in thymoma patients compared with patients with other types of cancer [9]. MG is frequently (up to 45% of cases) diagnosed when thymoma is detected [9]. As not all patients bearing a thymoma manifest an autoimmune disease, additional factors, genetic and/or environmental, predisposing to or promoting autoimmunity must be involved. Recently, Cufi and colleagues demonstrated that IFN type I overexpression together with activation of TLR3-mediated pathways are present in thymomas from MG patients; such findings suggest that MG might develop after a pathogen infection in thymoma patients with a genetic susceptible background [18], as it has been postulated for MG associated with thymic hyperplasia. Our previous study showed the presence of TLR4-expressing macrophages positive for poliovirus (PV) type 1 in 4 of 27 MG thymuses (14.8%), including two diffuse hyperplastic thymuses (or thymitis) and two thymomas, but not in 18 non-pathological control thymuses and 10 pathological thymuses (8 thymoma and 2 hyperplastic) from patients without MG, thus supporting the idea of a viral contribution to MG associated and not associated with thymoma [41]. Consistent with this hypothesis, Manca and colleagues detected gene sequences of the human T-cell leukemia virus I (HTLV-I) in a proportion (>55%) of MG thymuses with hyperplasia and thymoma [42], also suggesting that pathogen infections may be responsible for the chronic inflammatory state which characterizes MG thymus, making it prone to autoimmunity.

Among potential relevant micro-organisms, EBV is linked with different autoimmune diseases and cancers [21, 22], thus representing a major candidate to be involved in the pathogenesis of MG with thymoma. We previously demonstrated EBV persistence and reactivation in hyperplastic and involuted thymuses from MG patients, and suggested a contribution of EBV to immunological dysfunctions leading to MG [24, 25]. Unlike PV and HTLV-I, which were detected in a proportion of MG thymic tissues examined, but not in all [41, 42], we observed signs of EBV infection in B cells and plasma cells of all the hyperplastic and involuted MG thymuses investigated [24, 25], thus indicating that EBV may be a prominent feature of pathological thymuses from MG patients, likely playing a relevant role in B cell-mediated autoimmunity.

EBV involvement in thymoma, associated or not with MG, has never been unequivocally demonstrated. Since the 80's several studies attempted to identify EBV nucleic acids in MG and non-MG thymomas producing contrasting results [27–31], likely because of the different approaches employed and the use of virus detection techniques that would not be considered sufficiently sensitive today. Nevertheless, some of them provided evidence of EBV presence in some thymoma cases: i) McGuire et al. found EBV DNA in the 3 thymomas studied (2 with MG) [27]; ii) Chen et al. found EBV signals in 8 out of 21 thymic carcinomas with lymphoepithelioma-like morphology, a subtype not included in the present histological WHO classification, but they did not specify whether EBV-positive tumors were from MG patients [30]; iii) Takeuchi et al. analyzed 11 thymomas without MG and detected EBV-infected lymphocytes in one of them [31]. More recently, the search of potential pathogens in 18 MG thymomas was focused on human papillomavirus (HPV) and EBV; neither HPV nor EBV DNA were detected, but few details on EBV DNA detection protocol were provided [18]. Indeed, EBV detection methods are a matter of discussion since a long time: the main issue is the unequivocal identification of EBV in pathological tissues different from those obtained in EBV-driven lymphomas or acute EBV infected tissues/organs, characterized by high viral load and high rate of EBV replication [23]. As in our previous studies [24, 25], here we applied different molecular biology techniques, along with *in situ* hybridization, immunohistochemistry and confocal microscopy, to identify EBV presence in thymoma samples from MG and non-MG patients, reducing the risk of false-positives or -negatives. Using quantitative real-time PCR, we showed a significantly higher frequency of EBV DNA and EBER1 detection in MG than non-MG thymomas. EBER1 positivity (22/26) was higher than EBV DNA positivity (14/26) in MG thymomas, likely because multiple copies of EBER1 are present within EBV-infected cells [33]. The highest EBV DNA load values and EBER1 levels were observed in B2 and B2-mixed thymomas (Figure 1), the most often WHO types associated with MG [6, 14]. *In situ* hybridization confirmed molecular data by demonstrating the presence of cells positive for EBERs (both EBER1 and 2) in MG thymoma cases, but not in non-MG thymomas (Figure 2). As additional markers of EBV latency we analyzed EBNA2 transcript, whereas the early BZLF1 and the late gp350/220 lytic cycle transcripts were tested as reactivation markers. EBNA2 is the first gene to be expressed in infected cells *in vitro* and it is associated with the early stage of EBV infection [34]. We did not find EBNA2 transcripts in all, but one, MG thymomas, nor in non-MG cases, thus indicating absence of newly EBV infected cells in almost the totality (97.5%) of the examined tumoral tissues. Similarly, the early lytic EBV reactivation marker BZLF1 [35] was absent in all thymoma samples, whereas the late lytic gp350/220 marker was only found in the EBNA2-positive thymoma. This sample was from a MG patient [MG (+) T24, Table 1] positive for anti-AChR, -titin and -ryanodine receptor antibodies, having concomitant stiff-person syndrome associated with glutamic acid decarboxylase (GAD) antibodies, and transient myositis. Although rare, some cases of MG patients with stiff-person syndrome associated with anti-GAD antibodies have been previously described [43, 44]. Positivity of the MG (+) T24 patient for several autoantibodies, suggest a polyclonal B cell activation, likely as a consequence of EBV-induced autoimmunity [45].

In line with the *in situ* hybridization data on EBERs, cells positive for the latency protein EBNA1, indispensable for viral replication, genome maintenance and viral gene expression [46], were detected by immunohistochemistry in all the examined MG thymomas; rare EBNA1-positive cells were found in non-MG thymomas [MG (-) T6, 10 and 14] (Figure 3), the three samples resulted positive for EBV DNA and EBER1 transcript.

To analyze B cells and TECs as possible targets of EBV in thymoma, we firstly evaluated the degree of B cell infiltration in our thymoma samples, showing a higher number of B cells in MG versus non-MG thymomas, likely related to an increased expression of the B cell-attracting chemokine CXCL13 (Figure 3D), in line with previous reports [47–49]. Here, we have frequently found co-localization of the latency EBV proteins LMP1 and LMP2A with the CD20-positive B cells in MG thymomas, but not in the non-MG tumor samples (Figure 4). LMP2A was localized on rare TECs, whereas LMP1 was absent (Figure 5). Thus, our data suggest the association of EBV with B cell-mediated autoimmunity in MG, rather than with the neoplastic transformation of the thymic epithelial cells. This should imply that thymoma development might be the consequence of epithelial cell changes, not linked to EBV infection.

Different expression patterns of EBV latent genes determine the occurrence of EBV latency type I, II or III, each type being associated with distinct EBV-related diseases [23, 50]. EBERs as well as EBNA1 are expressed in all the latency types of EBV infection [50]. EBNA2 is expressed only during latency type III (known as growth program), which is typical of newly infected naïve B cells, lymphoproliferative disorders, and infectious mononucleosis [23, 50]. LMP1 and LMP2A are expressed during latency III and II (known as default program), with type II being observed in memory B cells and germinal center cells, Hodgkin and non-Hodgkin lymphoma, and nasopharyngeal carcinoma [23, 50, 51]. Finally, latency I (known as true latency) is characterized by EBNA1 and EBER expression only; it is typically observed in rare peripheral blood memory B cells and is associated with Burkitt's lymphoma [23, 50]. The viral expression profile emerging from our combined molecular and immunohistochemistry data on MG thymomas is indicative of EBV latency type II, since most of the samples were characterized by the expression of LMP1 and LMP2A and absence of EBNA2 transcript.

In contrast to alpha and beta herpesviruses, which cause diseases in humans during the lytic infection, but are essentially innocuous during the latency, most pathological conditions attributable to EBV are associated with the latent forms of infection [20]. EBV dysregulation of the host B cell system can lead to abnormal B cell survival and breakdown of B cell tolerance through mechanisms that involve: a) LMP1 and LMP2A expression, which are able to mimic activated CD40 and B cell receptor signaling [20]; b) induction of the anti-apoptotic protein Bcl-2 [52], known to be overexpressed in MG-associated thymomas [53]; c) synthesis of the B cell growth factor BAFF [54], known to be increased in serum and thymus of MG patients [55, 56]; and d) hypersensitivity to TLR stimulation [57]. We hence could argue that EBV is not an innocent bystander, but it may shape the local B cell repertoire, favor B cell survival and rescue potentially autoreactive B cells that would otherwise have undergone apoptosis. Increased surface expression of the EBV receptor CD21 in AChR-specific B cells of MG patients [58, 59] supports a possible contribution of EBV to autoreactive B cell activation and expansion in MG.

Most MG patients (n=17) were treated with corticosteroids (alone or in combination with azathioprine) before thymectomy (Table 1). However, we provided evidence of positivity for EBV markers also in thymoma from MG patients who had been treated only with acetylcholinesterase inhibitors [MG (+) T9, 15, 20, 23, 25] (Tables 1 and 2); moreover, no difference in EBV DNA load and EBER1 levels was observed between corticosteroids-treated and naïve patients, indicating that enrichment of EBV-harboring B cells in MG-associated thymomas is not strictly linked to the immunosuppressive therapy, as also observed in EBV-positive hyperplastic MG thymuses [24, 25].

In MG thymomas it has been recently reported a sustained IFN-mediated antiviral response and TLR3 overexpression [18]; our data on EBV detection in MG thymomas raised the question whether the virus itself may play a significant role in the inflammatory processes. Indeed, our data not only confirmed TLR3 up-regulation in MG thymomas versus normal thymuses, but also demonstrated that the expression of this receptor was higher in thymomas from MG patients compared with non-MG. Interestingly, transcriptional levels of TLR3 positively correlated with EBER1 levels in EBV-infected MG thymomas (Figure 6), thus suggesting TLR3 signaling activation by EBERs in MG-associated thymomas. Since TLR3 has been reported to induce thymic overexpression of the AChR-alpha subunit and promote an anti-AChR autoimmune response [60], the identification of a positive correlation between TLR3 and EBER1 supports the hypothesis of a contribution of EBV to B cell-mediated autoimmunity via TLR3 in MG thymomas. We can also suggest that EBV/EBER-driven TLR3 signaling may favor antiviral type I IFN production and inflammatory responses, which contribute to create a tumor microenvironment favoring the recruitment of peripheral B cells.

It is worth noting that EBV viral load estimated in our MG thymomas (less than 1,000 EBV DNA copies/10^6^ cells) was low if compared to that of a typical EBV-associated tumor, such as nasopharingeal carcinoma (~300,000 EBV copies/10^6^ cells) [32]. The relatively low viral load values in MG thymomas may be due to the absence of EBV reactivation in the neoplastic tissue and absence or low levels of EBV within the epithelial tumor cell population. However, irrespective of the EBV load values, total absence of positive signals in all the non-pathological control thymuses and the majority of non-MG thymomas examined, indicates that EBV presence in B cells of MG thymomas is relevant for thymoma-associated MG, an issue deserving to be addressed by further studies, particularly in view of the possible therapeutic implications that this discovery could have in the future.

Our overall findings show that EBV-infected tumor-infiltrating B cells are commonly present in MG thymomas, suggesting a contribution of EBV to B cell dysregulation and tolerance disruption in MG associated with thymic tumor.

## MATERIALS AND METHODS

### Patients, tissues and cell lines

The study included 26 MG thymoma (16 females and 10 males) and 14 non-MG thymoma (7 females and 7 males) patients. Mean age at thymectomy was 49.6 ± 12.4 for MG patients and 53.4 ± 17.0 years for non-MG patients. Patients’ clinical characteristics are summarized in Table 1. None of the non-MG thymoma patients had autoimmune diseases associated with thymoma. Non-pathological control thymuses were obtained from 6 cardiopathic patients during heart surgery (3 females, 3 males; mean age at surgery 30.8 ± 15.8 years). The study was approved by the Ethic Committee of the Neurological Institute ‘Carlo Besta’, and each patient provided written informed consent for thymectomy and use of thymus specimens for research purposes. Molecular and immunohistochemistry analyses were performed on snap-frozen fragments of thymomas and non-pathological control thymuses; *in situ* hybridization to detect EBER1 and 2 was performed on available formalin-fixed paraffin-embedded thymoma tissue blocks and adjacent non-tumoral thymic tissue blocks. TEC primary cultures were established from available freshly isolated thymoma fragments from 3 MG patients [MG (+) T11, 14 and 16] (Table 1), as previously described [61], and their purity (> 90%) was checked by anti-cytokeratin (1:100, clone MNF116, Dako, Glostrup, Denmark) immunofluorescence staining followed by Cy2-conjugated goat anti-mouse IgGs (Jackson Immunoresearch Laboratories, West Grove, PA, USA). As positive controls, snap-frozen thymic sections of a patient with an EBV-positive mediastinal B cell lymphoma and of a patient with a classical EBV-associated Hodgkin's lymphoma were included in molecular and immunohistochemistry analyses, whereas snap-frozen sections of a solitary pleural fibrous tumor were analyzed as negative control.

EBV-positive lymphoblastoid Namalwa and JY cell lines [62, 63], and EBV-negative human Jurkat T cell line [64] were purchase from Sigma Aldrich SRL (Milan, Italy) and cultured in RPMI 1640 (Euroclone, Pero, Italy) with 10% fetal bovine serum (Thermo Fisher Scientific, Waltham, MA), 2 mM sodium pyruvate, 2 mM L-glutamine, and 100 U penicillin/streptomycin (all from Euroclone) at 37°C in 5% CO_2_. DNA and RNA were extracted from each cell line and used as positive (Namalwa and JY) or negative (Jurkat) controls in molecular analyses.

### Real-time PCR for EBV DNA load quantification

DNA was extracted from thymoma and control tissues and cell lines using the Nucleospin Tissue kit (Macherey-Nagel, Düren, Germany). A Taqman multiplex real-time PCR was performed to simultaneously detect the EBV Pol and the cellular β2m genes as previously reported [32]. Briefly, amplification was performed in a total volume of 25 μl containing 0.5 μg of DNA, 1X Taqman Universal PCR Master Mix (Thermo Fisher Scientific), 0.18 μM forward and reverse Pol primers, 0.1 μM FAM-labeled Pol probe, 0.09 μM forward and reverse β2m primers, and 0.08 μM VIC-labeled β2m probe. Primer and probe sequences were reported in [32]. Following one step at 50°C for 2 min and one at 95°C for 10 min, 50 cycles of amplification at 95°C for 15 sec and 60°C for 1 min were carried out by a ViiA7 Real-time PCR System (Thermo Fisher Scientific). Pol and β2m standard curves, used to determine the number of EBV genomes and the cell input in each sample, were generated from the amplification of serial dilutions of the Namalwa cell DNA containing 1 to 10^5^ EBV genome copies, assuming that diploid Namalwa cells carry two EBV genomes. The detection limit of the assay was estimated as being two EBV genomes per reaction (Supplementary Figure 1). The samples, including no template control, were analyzed in duplicate and considered negative for Pol Ct value higher than 40 cycles.

### Molecular analyses for EBV and innate/adaptive immune transcripts

Total RNA was extracted from tissues, TECs and EBV-positive and -negative control cell lines using the TRIzol method and treated with DNAse I (Thermo Fisher Scientific).

Real-time PCR for the detection of the EBV latency marker EBER1 was performed on RNA using the TaqMan Fast Virus 1-Step Master Mix (Thermo Fisher Scientific), that allows reverse transcription and PCR all in one reaction, as previously described [25]. Primer and probe sequences for EBER1 were previously reported [65]. Human 18S gene was analyzed in each sample using a specific Taqman gene expression assay and served as endogenous control for data normalization using the formula 2^-ΔCt^× 100. Supplementary Figure 2 shows an example of amplification plot obtained by real-time PCR analysis of EBER1 and 18S in tissue samples resulted positive and negative for EBER1.

For detection and quantification of latent EBNA2, early lytic BZLF1, late lytic gp350/220, CD19, marker for B cells, CXCL13, B-lymphocyte chemoattractant, TLR3, TLR7 and TLR9 transcripts, random-primed cDNA was prepared from the DNase-treated RNA samples using Superscript VILO cDNA synthesis kit (Thermo Fisher Scientific).

For EBNA2 and gp350/220, real-time PCR reactions were performed in duplicate in a final volume of 20 μl containing 1X SYBR Green mix (Thermo Fisher Scientific) and 1 μM of each primer, whose sequence was as previously described [34, 35]. As endogenous control, the human 18S transcript was amplified from each cDNA preparation using the following primers: forward 5′-GTCTGTGATGCCCTTAGATG-3′; reverse 5′-AGCTTATGACCCGCACTTAC-3′. Cycling conditions were as follows: after one step at 50°C for 2 min and one at 95°C for 10 min, 50 cycles of amplification at 95°C for 15 sec and 60°C for 1 min were carried out by the ViiA7 Real-time PCR System, followed by a melting curve stage at 95°C for 15 sec, 60°C for 1 min and 95°C for 15 sec.

BZLF1 was analyzed by nested PCR, as previously described [24]. As control of RNA integrity and retrotranscription efficiency, the β-actin gene was amplified from each cDNA preparation. The PCR products were separated by electrophoresis on 2% agarose gel stained with SERVA DNA stain clear G (SERVA, Heidelberg, Germany) and visualized under ultraviolet light using the Molecular Imager Gel Doc System (Bio-Rad Laboratories, Hercules, CA).

Transcriptional levels of CD19, CXCL13, TLR3, TLR7 and TLR9 were quantified in duplicate reactions using specific TaqMan gene expression assays (Thermo Fisher Scientific), and expressed as relative values (2^-ΔCt^ x 100) normalized to 18S transcript.

### *In situ* hybridization for EBERs

*In situ* hybridization was performed using the Epstein-Barr virus (EBER) PNA Probe/Fluorescein and the PNA ISH detection kit (Dako) to detect EBERs on paraffin-embedded thymoma and non-tumoral adjacent thymic tissue sections from 9 MG thymomas [MG (+) T1, 5, 9, 10, 11, 14, 15, 16, and 26] and 5 non-MG thymomas [MG (-) T2, 5, 7, 9, 13]. A sense probe was used as negative control. Synchronous pleural metastases were also analyzed in 2 of the 5 non-MG thymomas. Images were digitally acquired with the Aperio ScanScope system and visualized using the ImageScope v11.2.0.780 software (Aperio, Nikon GmbH, Germany).

### Immunohistochemistry analysis of latent EBV protein EBNA1

Immunohistochemistry was performed on 10 μm-thick frozen sections from 12 MG [MG (+) T3, 7, 8, 11, 16, 17, 19, 20, 21, 22, 23, and 24] and 7 non-MG [MG (-) T1, 6, 10, 11, 12, 13, and 14] thymomas, 3 control thymic tissues, the B cell lymphoma, the Hodgkin's lymphoma, and the pleural tumor. Sections were fixed in 4% paraformaldehyde (PFA) for 10 min, incubated in 1.5% hydrogen peroxide in methanol for 15 min, to eliminate endogenous peroxidase activity and simultaneously permeabilize cell membrane, and in 5% bovine serum albumin (BSA) for 1 h, to block non-specific binding sites. Then, sections were immunostained with primary antibodies specific for the B cell marker CD20 (1:300, clone L26, Dako, Catalog Number: M0755), and the latent EBNA1 (1:20, Acris Antibodies GmbH, Herford, Germany, Catalog Number: BM1083) over-night at 4°C. For EBNA1 detection, antigen retrieval was obtained by microwave heating of sections in citrate buffer (10 mM, pH 6.0) before incubation with primary antibody. Secondary labelling was performed with HRP anti-mouse antibody (Dako) followed by incubation with 3,3'-diaminobenzidine (DAB; Dako) and hematoxylin counterstaining. Negative controls included IgG isotype controls. Images were digitally acquired with the Aperio ScanScope system and visualized using the ImageScope v11.2.0.780 software (Aperio, Nikon GmbH, Germany). For each thymus, a whole histological section (at least 15 mm^2^) was analyzed. EBNA1-positive cells were counted and their number was normalized to the total tissue surface analyzed, expressed as mm^2^.

### Double immunofluorescence analysis of latent EBV proteins LMP1 and LMP2A

Ten-μm frozen sections from 10 MG [MG (+) T3, 4, 7, 11, 12, 14, 17, 20, 22, 24] and 5 non-MG [MG (-) T1, 6, 11, 12, 14] thymomas, 3 control thymic tissues, the B cell lymphoma, the Hodgkin's lymphoma, and the pleural tumor, were fixed in 4% PFA for 10 minutes, incubated in cold methanol for 10 min to allow cell membrane permeabilization and then in 5% BSA for 1 hour for blocking non-specific binding sites. The sections were stained over-night at 4°C with primary antibodies for: CD20, a B cell marker (1:300, Dako, or 1:2, Abcam, Cambridge, UK, Catalog Number: ab73095), cytokeratin, TEC marker (1:100, Dako, Catalog Number: M0821; or 1:100, Novocastra, Milan, Catalog Number: NCL-CKp), LMP1 (1:2, Dako, Catalog Number: IRF753) and LMP2A (1:300, Abcam, Catalog Number: ab59026), two EBV latent membrane proteins. Secondary antibodies were: Cy2-conjugated goat anti-mouse IgG, Cy3-conjugated goat anti-rabbit IgG and Cy3-conjugated goat anti-rat IgG (Jackson Immunoresearch Laboratories). Nuclei were stained with 4,6-diamidino-2-phenylindole, dihydrochloride (DAPI; Thermo Fisher Scientific). As negative control, primary antibodies were omitted or replaced with isotype-specific mouse, rat or rabbit IgG. Fluorescence images were captured by a confocal microscope system (C1 laser scanning Nikon) and analyzed using Image J software (version 1.43u). In MG thymomas, CD20 positive and CD20/LMP2A double positive cells were counted in at least 4 adjacent fields per section at 60× magnification.

### Statistical analysis

Statistical analysis was performed using R statistical software (version 3.0.2.) (www.r-statistics.org). Data distribution was tested via Shapiro-Wilk test and non-parametric data (p<0.05) were analyzed by Kruskal-Wallis test with Bonferroni post-hoc test for multiple comparisons, or by Mann-Whitney test for comparison of two groups. For comparison of EBER1 detection frequencies in MG versus non-MG thymomas, the Chi-square test was used and odds ratio (OR) was calculated. The non-parametric Spearman correlation test was applied to evaluate possible correlation between TLR3 and EBER1 transcript levels in MG thymomas. All tests were two-tailed, and a p-value of <0.05 was considered statistically significant.

## SUPPLEMENTARY MATERIALS FIGURES AND TABLES


