# Supplementary material for: Epstein-Barr virus in tumor-infiltrating B cells of myasthenia gravis thymoma: an innocent bystander or an autoimmunity mediator?
Source: Oncotarget. 2017 Sep 8;8(56):95432–49. doi: 10.18632/oncotarget.20731 (PMC5707033; doi:10.18632/oncotarget.20731)
Supplement: Supplementary file 1 [file oncotarget-08-95432-s001.pdf]

Epstein-Barr virus in tumor-infiltrating B cells of myasthenia gravis thymoma: an innocent bystander or an autoimmunity mediator?

SUPPLEMENTARY MATERIALS

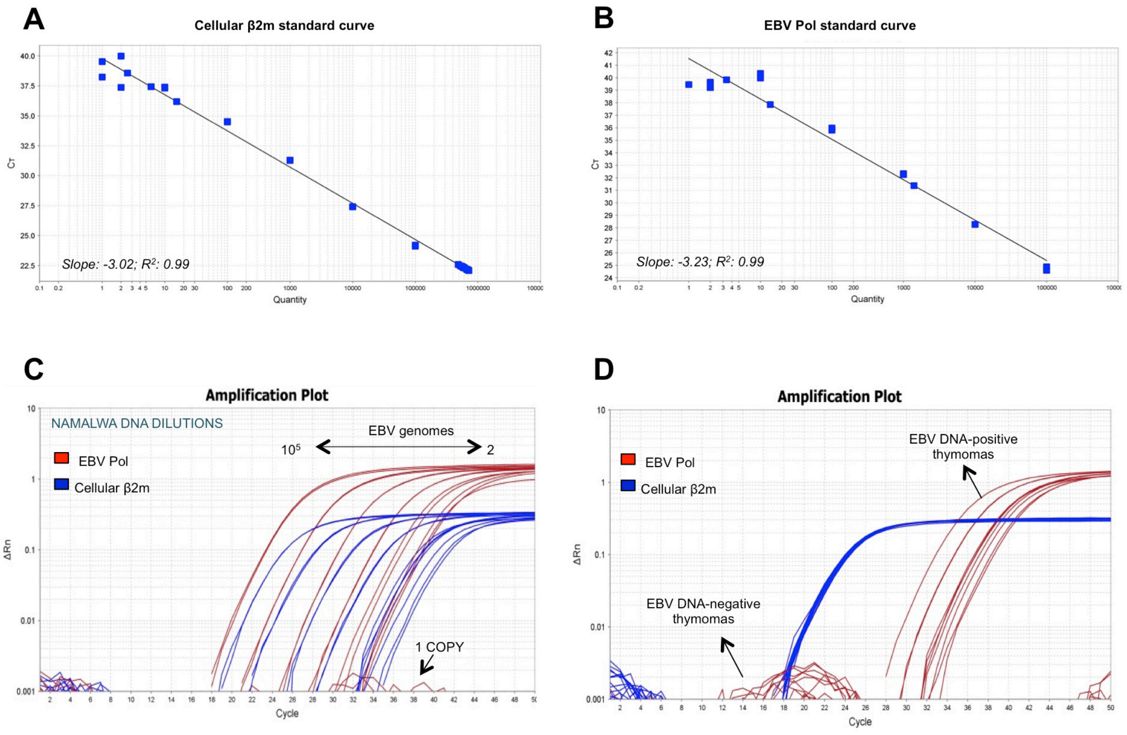

Supplementary Figure 1: Detection and quantification of EBV genome by real-time PCR. Calibration curves generated by amplifying serial dilutions of Namalwa DNA containing 10^5, 10^4, 10^3, 10^2, 10, 2 and 1 EBV genome copies using primer and probes specific for the cellular beta 2 microglobulin (β2m) **(A)** and the EBV DNA polymerase (Pol) **(B)** gene sequences in duplex real-time PCR reactions. For each sample, Ct values were plotted against the initial number of EBV genome copies (Quantity). The detection limit of the assay was two EBV genomes per reaction. **(C)** Amplification plot showing the changes in FAM (EBV Pol) and VIC (cellular β2m) fluorescent intensity (ΔRn) plotted against cycle number during amplification of Namalwa DNA serial dilutions. **(D)** Amplification curves obtained by amplifying EBV Pol and cellular β2m sequences in thymoma samples positive and negative for EBV DNA.

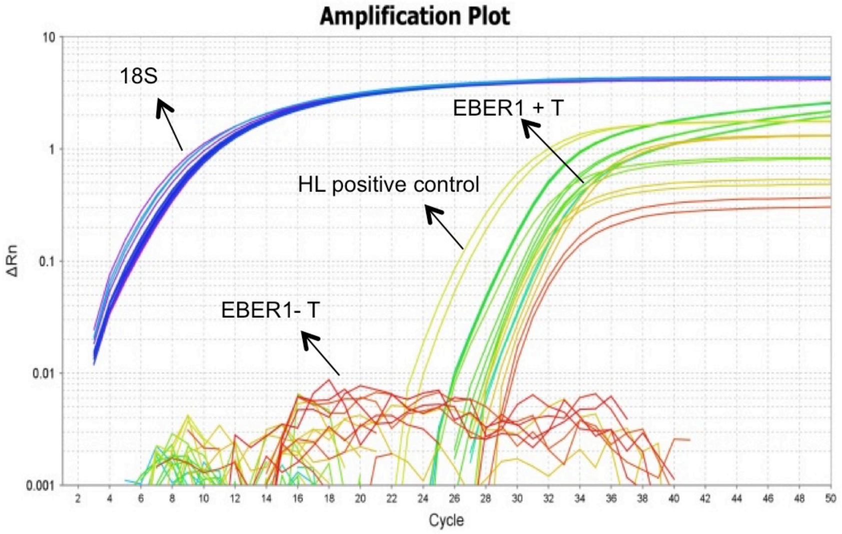

Supplementary Figure 2: Detection of the latent EBV-encoded small nuclear RNA 1 (EBER1) by real-time PCR. Representative EBER1 and 18S amplification curves of thymomas, in which EBER1 was detected (EBER1+ T) or undetected (EBER1- T). HL, positive control: amplification curve obtained by amplifying EBER1 in cDNA from Hodgkin's lymphoma.

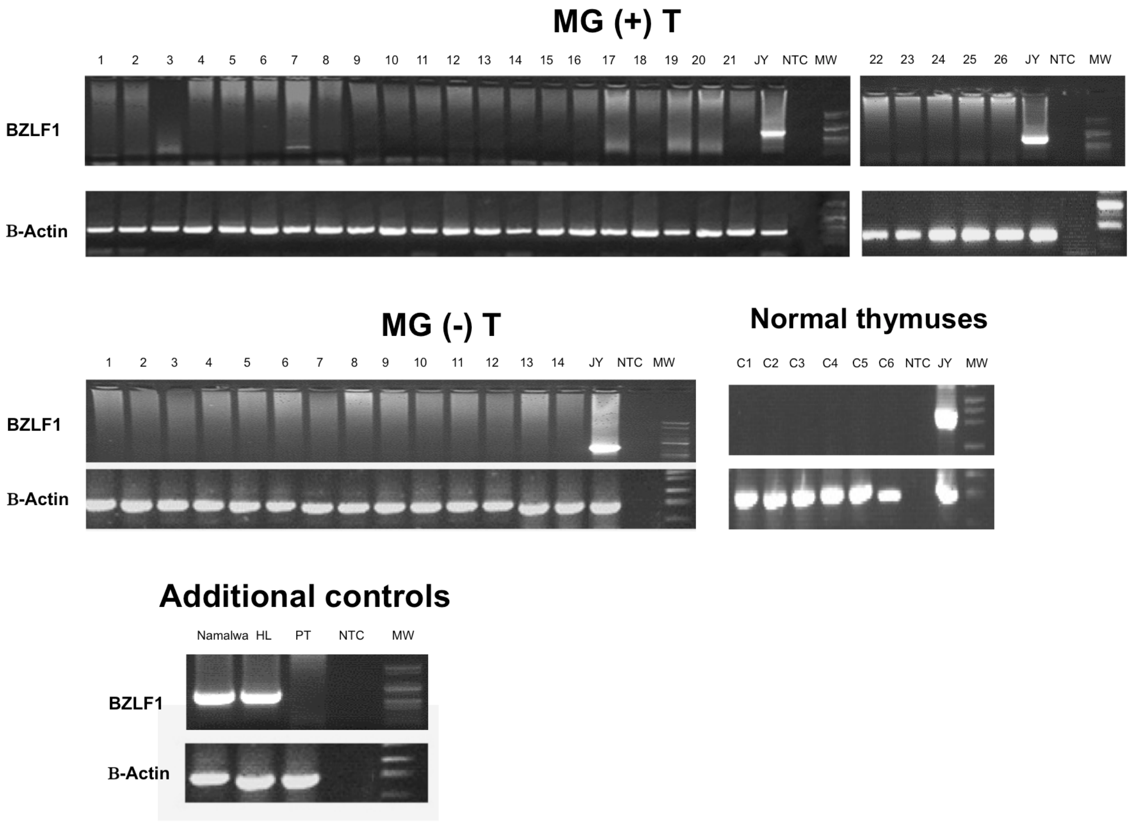

Supplementary Figure 3: Analysis of BZLF1 transcript in MG and non-MG thymomas by nested PCR. Gel electrophoresis of amplification products of lytic BZLF1 transcript (442 bp) from MG (+) (n=26) and MG (-) (n=14) thymomas, and control thymuses (C1-C6). As negative controls, cDNA from pleural fibrous tumor (PT) and a no-template-control (NTC) were amplified. As positive controls, cDNAs from the EBV-positive lymphoblastoid cell lines JY and Namalwa, and from Hodgkin's lymphoma (HL) were amplified. β-actin (234 bp) served as control for RNA integrity and retrotranscription efficiency. MW: DNA molecular weight marker (size range: 154–2,176 bp), mixture of pBR328 DNA, cleaved with *Bgl I*, and pBR328 DNA, cleaved with *Hinf I*.

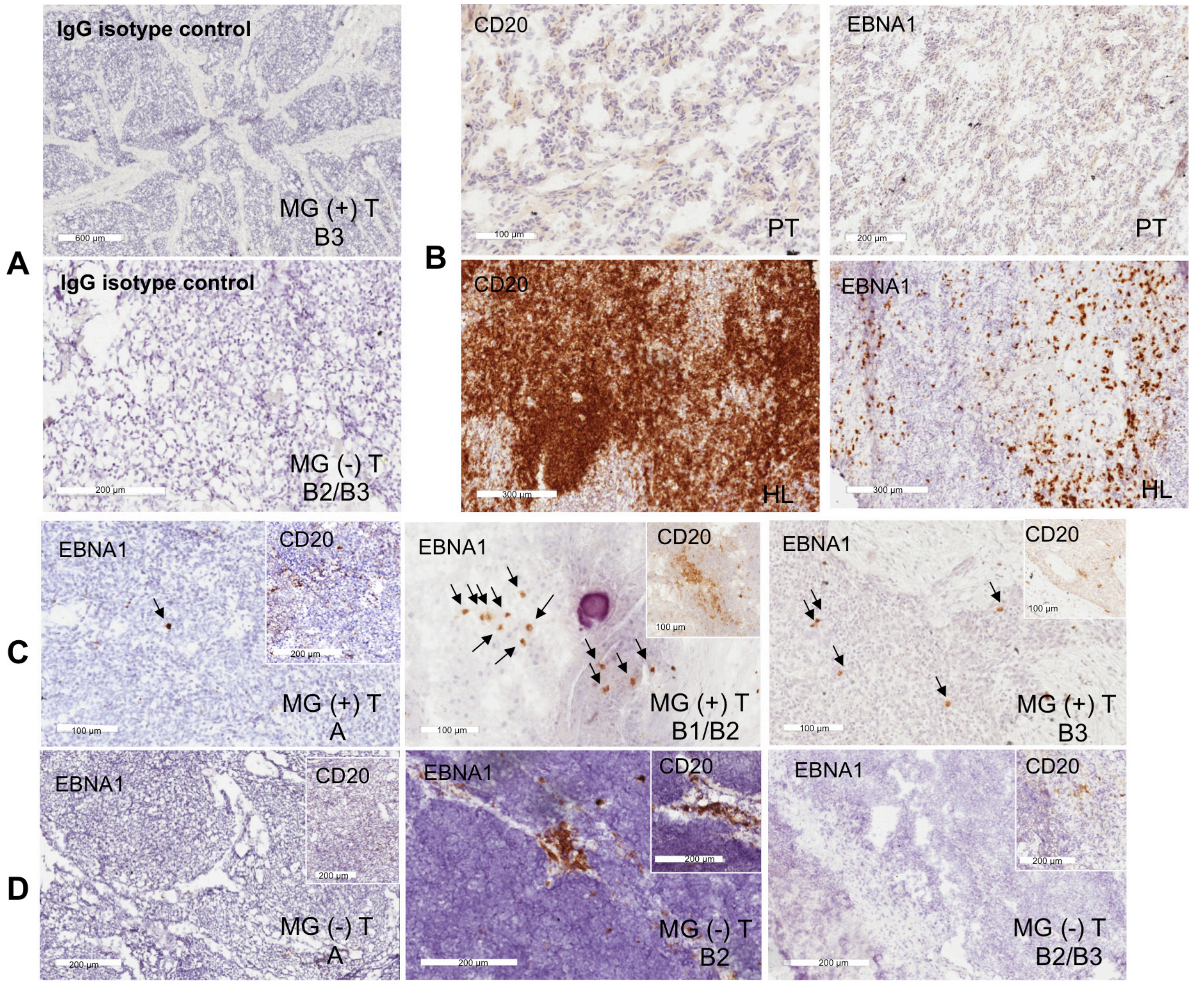

Supplementary Figure 4: Immunohistochemistry analysis of CD20 and EBNA1 in MG thymomas, non-MG thymomas and controls. **(A)** Absence of non-specific immunostaining in MG (upper panel) and non-MG (lower panel) thymomas incubated with isotype-specific non-immune IgG2 as control of EBNA1 immunostaining specificity. **(B)** Upper panels show absence of positivity for CD20 and EBNA1 in pleural fibrous tumor (PT); lower panels show the presence of numerous CD20-positive and EBNA1-positive cells in thymic sections of a non-MG patient with classical EBV-associated Hodgkin's lymphoma (HL). **(C)** Immunohistochemistry analysis showing the presence of EBNA1-positive cells in MG thymomas. Panels from left to right correspond to immunostaining images of thymoma sections from the following patients: MG (+) T3 (type A), T23 (type B1/B2), and T22 (type B3). **(D)** Immunohistochemistry analysis showing absent or rare EBNA1-positive cells in non-MG thymomas. Panels from left to right correspond to immunostaining images of thymoma sections from the following patients: MG (-) T1 (type A), T6 (type B2), T12 (B2/B3). In each (C) and (D) panel, the inset displays a section of the thymoma reported in the main panel stained to reveal CD20-positive cells.

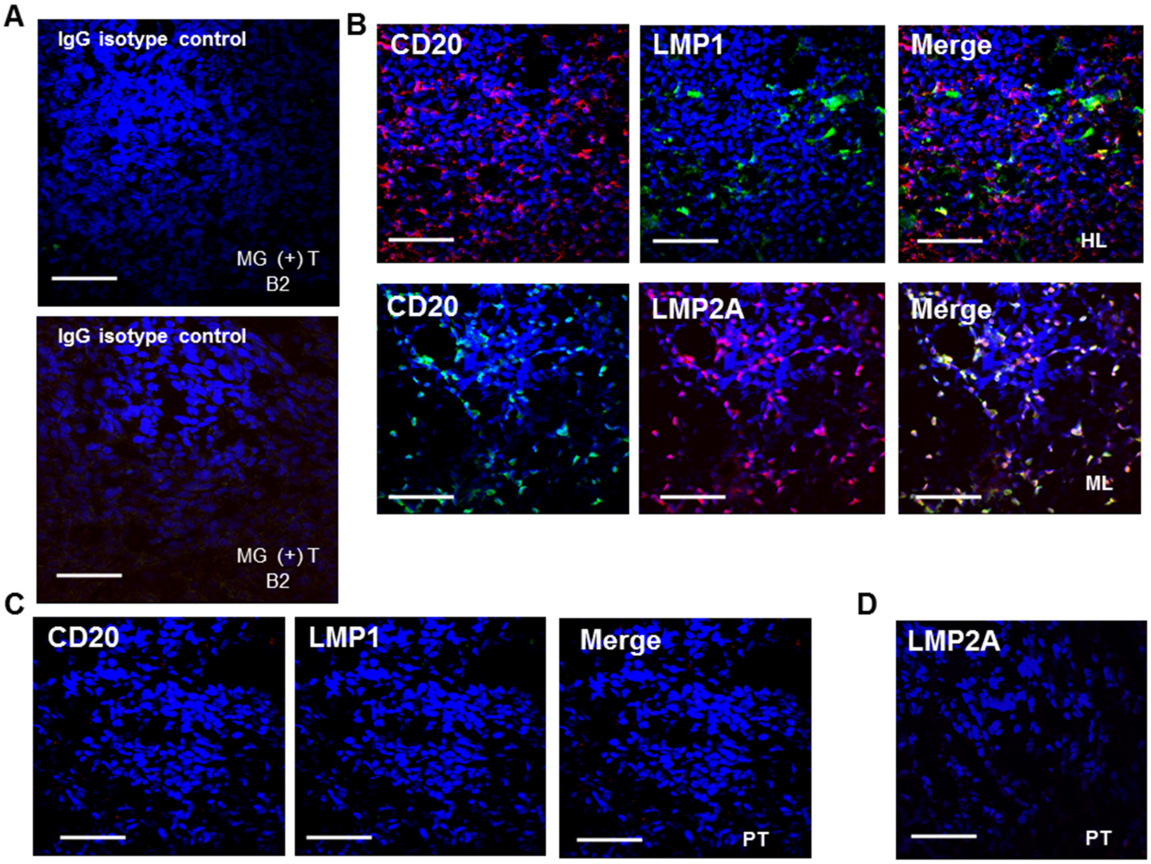

Supplementary Figure 5: Negative and positive controls in double immunofluorescence analysis to show latent LMP1 and LMP2A EBV protein expression in B cells or thymic epithelial cells of thymomas. **(A)** Representative image showing absence of non-specific immunostaining in thymoma sections [MG (+) T17 (type B2)] incubated with isotype-specific non-immune IgGs: mouse IgG1 followed by secondary labeling with Cy2-coniugated goat anti-mouse IgG, as negative control for LMP1 staining (upper panel); rat IgG2 followed by secondary labeling with Cy3-coniugated goat anti-rat IgG, as negative control for LMP2A staining (lower panel). **(B)** Representative image showing a thymic section from a non-MG patient with classical EBV-associated Hodgkin's lymphoma (HL) labeled for CD20 (red), marker of B cells, and LMP1 (green), upper panels; representative image showing a thymic section from a non-MG patient with mediastinal B cell lymphoma (ML) labeled for CD20 (green) and LMP2A (red), lower panels. **(C)** Section of a pleural fibrous tumor (PT) labeled for CD20 (red) and LMP1 (green). **(D)** Single staining for LMP2A (red) of a PT section. Blue staining in A to C panels shows DAPI-positive nuclei. Magnification bars: 50 µm.

Supplementary Table 1: Detection of EBV-encoded small RNAs (EBERs) by *in situ* hybridization in MG and non-MG thymomas, in relationship to EBV DNA load and EBER1 transcript detection

Thymoma sample	WHO histological type	EBV DNA load[a] by real-time pcr	Detection of EBER1 transcript by real-time PCR	Detection of EBERs-positive cells by ISH
MG (+) T1	A	9.6	+	Und
MG (+) T5	AB	Und	+	Und
MG (+) T9	AB	Und	+	Und
MG (+) T10	B1	Und	+	Und
MG (+) T11	B1	7.8	+	+
MG (+) T14	B2	391.0	+	+
MG (+) T15	B2	Und	+	+
MG (+) T16	B2	Und	+	+
MG (+) T26	B2/B3	226	+	+
MG (-) T2	AB	Und	Und	Und
MG (-) T5	B1	Und	Und	Und
MG (-) T7	B2	Und	Und	Und
MG (-) T9	B3	Und	Und	Und
MG (-) T13	B2/B3	Und	Und	Und

MG (+) T: thymomas from MG patients. MG (-) T: thymoma from patients without MG. For each patient, a snap-frozen thymoma fragment was used for real-time PCR analyses specific for EBV DNA and EBV-encoded small RNA (EBER) 1 transcript, and a paraffin-embedded tissue fragment was used for ISH *in situ* hybridization (ISH) for EBERs.
[a]EBV DNA load expressed as number of EBV genome copies per 1 million cells of thymoma tissue.
+: Detected; Und: undetected.
